# Common gamma chain (γc) cytokines differentially potentiate TNFR family signaling in antigen-activated CD8^+^ T cells

**DOI:** 10.1186/s40425-014-0028-y

**Published:** 2014-09-16

**Authors:** Michael J McNamara, Melissa J Kasiewicz, Stefanie N Linch, Christopher Dubay, William L Redmond

**Affiliations:** 1 Robert W. Franz Cancer Research Center, Earle A. Chiles Research Institute, Providence Portland Medical Center, 4805 NE Glisan St., 2 N35, Portland, 97213, OR, USA

**Keywords:** CD8+ T cell, Common gamma chain cytokines, IL-2, TNFR, OX40, Immunotherapy

## Abstract

**Background:**

Several members of the common gamma chain (gc) cytokine family are already approved (IL-2) or actively being developed as vaccine adjuvants and cancer immunotherapies. Studies have indicated that co-administration of gc cytokines may enhance the efficacy of immunotherapies that function via direct activation of co-stimulatory T cell receptors. To define the specific influence of gc cytokines on the co-stimulatory capacity of CD8^+^ T cells and identify combinations with synergistic potential, we investigated the direct impact of gc cytokines on the differentiation and transcriptional profile of recently antigen-primed CD8^+^ T cells.

**Methods:**

Naïve CD8^+^ T cells were activated with peptide-pulsed APCs. After 48 hours, CD8^+^ T cells were harvested and re-cultured in media supplemented with IL-2, IL-4, IL-7, IL-15 or IL-21. After 24 hours, cells were analyzed by cytokine bead array, flow cytometry, and mRNA micro-array. Gene networks responsible for specific CD8^+^ T cell functions were constructed through literature-meta review and publicly available annotation databases. Gene expression data from the experimental groups was imported into this network to visualize the impact of each gc cytokine on the functional polarization of recently-activated CD8^+^ T cells.

**Results:**

Among the gc cytokines, IL-2 induced the greatest increase in the expression of co-stimulatory receptors in recently-activated CD8^+^ T cells. IL-2 increased significantly expression of 4-1BB, GITR, ICOS and OX40, at both the transcriptional and protein level. IL-2 also drove the greatest increase in cellular proliferation and the most robust shift towards a pro-survival phenotype, compared with the other gc cytokines. Both IL-4 and IL-21 enhanced expression of cytotoxic effector proteins, but drove distinct phenotypic polarizations, Th2/Tc2 and NK-like, respectively.

**Conclusions:**

Overall, these observations suggest that among gc cytokines, IL-2 may be uniquely capable of synergizing with therapeutic strategies that combine immunization with agonists of co-stimulatory T cell receptors. Previous studies have shown that the timing of IL-2 treatment relative to immunization plays a key role in defining the CD8^+^ T cell response, and the findings from this study indicate that administration of exogenous IL-2 shortly after the initial antigen-priming event has concluded may augment the receptivity of these cells to subsequent TNFR co-stimulation.

## 1 Background

The common gamma chain (γc) receptor family of cytokines has a central role in the regulation of both innate and adaptive immunity. A subset of type I cytokines, the γc family, includes interleukin-2 (IL-2), IL-4, IL-7, IL-9, IL-15 and IL-21. Each of these cytokines signals via a multimeric receptor complex that incorporates the common gamma chain receptor, CD132. The γc cytokines are especially important in lymphocyte development, where they influence polarization, proliferation and survival [1]. Because of this influence, the therapeutic potential of γc family cytokines has been investigated in the context of numerous biomedical applications, notably cancer immunotherapy [2],[3]. High dose IL-2 therapy, which drives an expansion of lymphocyte populations, is currently approved by the FDA for the treatment of metastatic melanoma and renal cancer [4]. The remaining γc cytokines, with the exception of IL-9, are at various stages of pre-clinical development for immunotherapy applications [1]. Beyond their utility as monotherapies, ongoing research has indicated that the combination of exogenous γc cytokines with agonistic monoclonal antibodies that target TNFR family receptors on T cells can enhance anti-tumor immunity in multiple cancer models [5],[6]. Thus, a thorough understanding of how γc cytokines modulate and potentiate TNFR signaling in activated CD8^+^ T cells is important for the development of better combinatorial cancer immunotherapies.

Following TCR stimulation, CD8^+^ T cells undergo clonal expansion, acquire effector functions and can be polarized in multiple dimensions; cytokine production vs. cytolytic activity, short-lived vs. long-lived, co-stimulatory vs. co-repressive, peripheral vs. central homing, etc. [7],[8]. From a general perspective, CD8^+^ T cells can be polarized into Tc1, Tc2, and Tc17 cell populations, which parallel the polarization of CD4^+^ T cells into Th1, Th2, and Th17 cell populations, respectively [9]. The γc cytokines appear to be critical determinants of how CD8^+^ T cells progress through these developmental pathways [10]. Their receptor complexes utilize shared subunits that transduce overlapping signals via the JAK–STAT pathway and the differential activation of STAT pathways likely contributes to the distinct effects induced by each γc cytokine [11]. For leukocytes in general, IL-2, IL-7, and IL-15 have been shown to preferentially activate STAT5, while IL-4 and IL-21 preferentially activate STAT6 and STAT3, respectively . For CD8^+^ T cells specifically, recent work has suggested that IL-4 and IL-21 signal largely through STAT3, with minimal activation of STAT6 [1]. As a result of these unique signal transduction pathways, each γc cytokine likely has a distinct impact on the development and function of recently activated CD8^+^ T cells, and by extension, their capacity to respond to various signals via the TNFR family of cell surface receptors.

Ligation of co-stimulatory T cell receptors, such as OX40 (Tnfrsf4, CD134), 4-1BB (Tnfrsf9, CD137) and GITR (Tnfrsf18, CD357), with agonist mAbs has been shown to augment the anti-tumor immune response in numerous cancer models [12]. Exposure to IL-2 up-regulates OX40 expression on effector CD8^+^ T cells and IL-2 therapy has been shown to enhance the therapeutic efficacy of an agonist anti-OX40 mAb [6]. The potential synergy between γc cytokines and T cell stimulating mAbs offers the prospect of significantly improved therapeutic outcomes. However, the molecular mechanisms by which γc cytokines influence the TNFR-dependent signaling networks of T cells remain largely unexplored. While the systemic effects of administering exogenous γc cytokines have been investigated in animals and humans, the complexity, redundancy, and cross-talk within the γc signaling network presents a challenge to defining the *direct* effects of each γc cytokine on specific lymphocyte subpopulations. The purpose of this study was to investigate the direct influence of γc family cytokines on the differentiation and polarization of freshly antigen-primed CD8^+^ T cells in an effort to better define the functional relationship between γc cytokines and TNFR-mediated co-stimulation in this population.

## 2 Results

### 2.1 The γc cytokines differentially impact the proliferation and survival of recently activated CD8^+^ T cells

**Figure 1:**
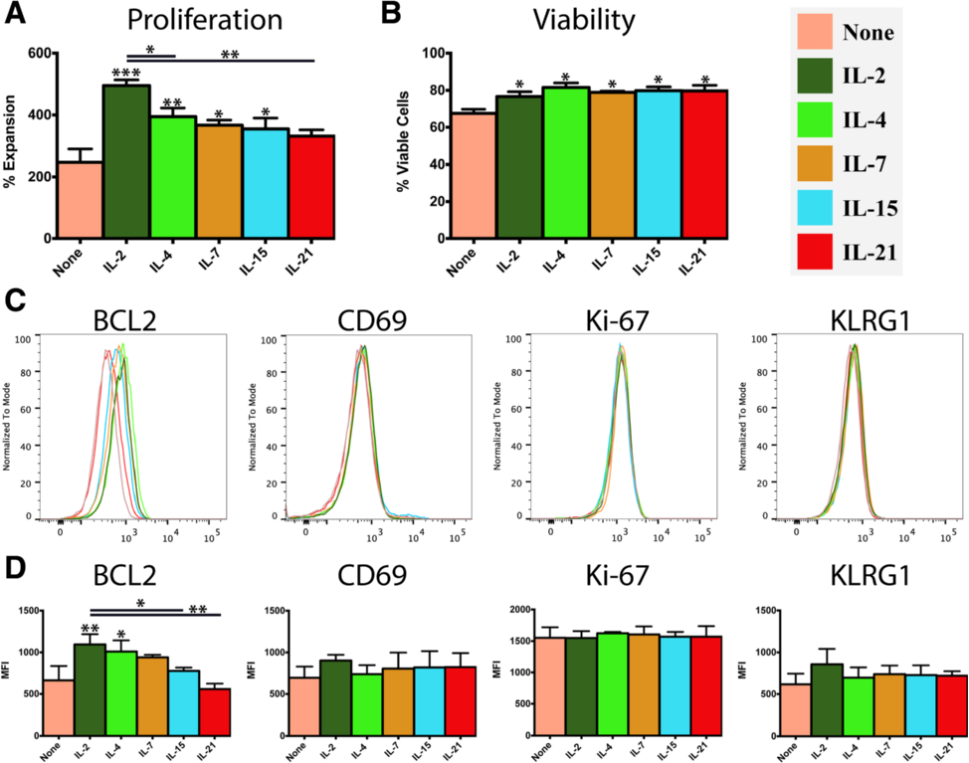
**Influence of γc cytokines on proliferation, survival and activation state of recently-activated CD8**^**+**^**T cells.** Purified naïve OT-I CD8^+^ T cells (1x10^6^/ml) were stimulated with peptide-pulsed APCs (6x10^6^/ml). After 48 hours, OT-I T cells were harvested, re-purified and re-cultured (5x10^5^/ml) in the presence of either plain media or media supplemented with IL-2, IL-4, IL-7, IL-15 or IL-21 (100 ng/ml). After 24 hours, cells were harvested and the analyzed. **A)** Expansion of OT-I CD8^+^ T cells in response to each treatment condition. **B)** Percentage of viable cells in each condition, as determined by Trypan Blue exclusion. **C**, **D)** Flow cytometry analysis of T cell markers associated with activation state, proliferation and exhaustion. Data are generated from 3 biological replicates, histograms reflect a single representative biological replicate and the bar graphs depict the mean of replicates +/−SD (n = 3). Unless otherwise noted, significance reflects the difference between the treatment group and the media only control. *P < 0.05, *P < 0.01, ***P < 0.001.

In this experimental model, activated CD8^+^ T cells are purified shortly after antigen-priming and cultured in the presence of individual γc cytokines to define their direct effect on short term differentiation of this T cell population. A significant increase in cellular proliferation, relative to untreated controls, was observed in response to treatment with all of the γc cytokines except IL-21 (Figure 1A), with IL-2 driving the most robust expansion. The proportion of viable cells was largely consistent in each treatment group, and all of the treatment groups had a significantly higher proportion of viable cells than the untreated control (Figure 1B). The differences in proliferation were paralleled at the protein level by the anti-apoptotic transcription factor, BCL2, which was elevated in all of the treatment groups and observed at greatest abundance in response to IL-2. In contrast, several markers of T cell activation (Ki-67, CD69 and KLRG1) were detected at levels that were consistent across all experimental groups (Figure 1C-D).

### 2.2 IL-2 uniquely enhances the expression of several co-stimulatory TNFR family receptors on activated CD8^+^ T cells

**Figure 2:**
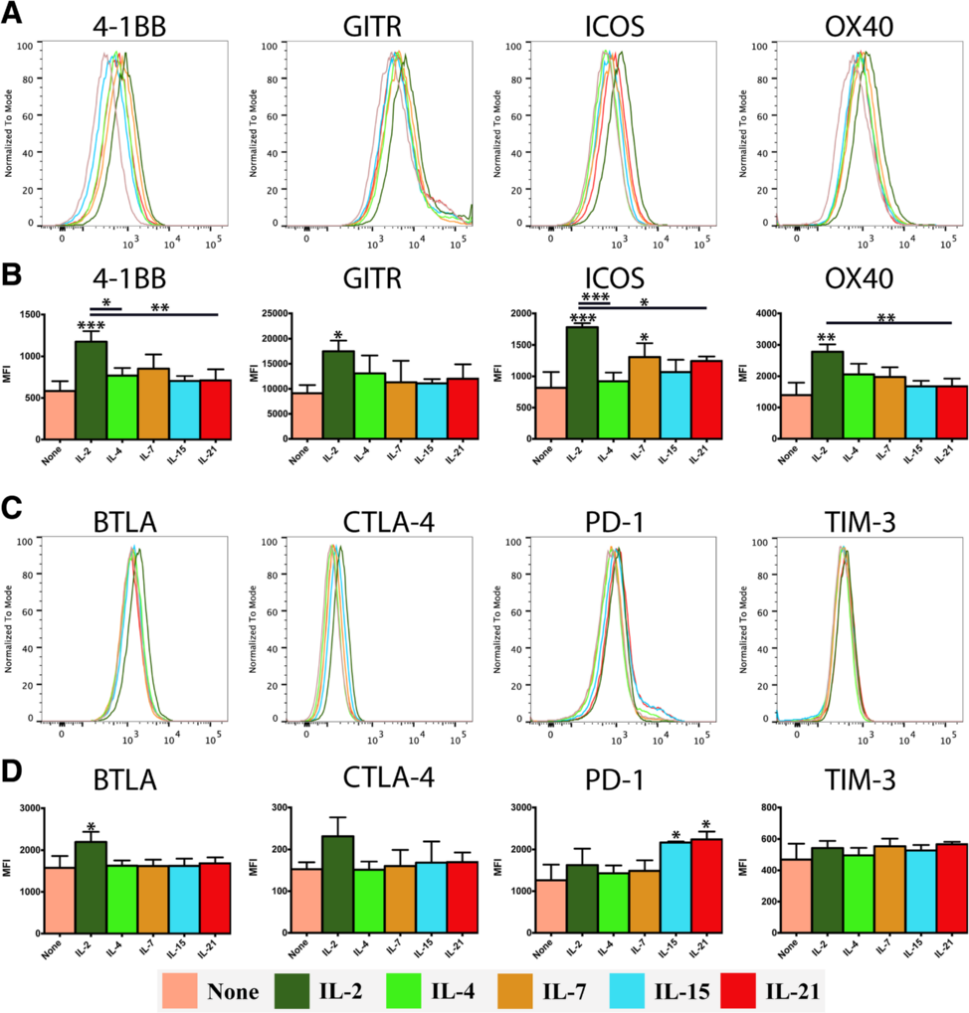
**Effect of γc cytokines on the expression of co-stimulatory and co-repressive receptors by recently-activated CD8**^**+**^**T cells.** Purified naïve OT-I CD8^+^ T cells (1x10^6^/ml) were stimulated with peptide-pulsed APCs (6x10^6^/ml). After 48 hours, OT-I T cells were harvested, re-purified and re-cultured (5x10^5^/ml) in the presence of either plain media or media supplemented with IL-2, IL-4, IL-7, IL-15 or IL-21 (100 ng/ml). After 24 hours, cells were harvested and the analyzed by intracellular cytokine staining and flow cytometry. **A**, **B)** Expression of co-stimulatory CD8^+^ T cell receptors. **C**, **D)** Expression of co-repressive and suppressive CD8^+^ T cell receptors. Data are generated from 3 biological replicates, histograms reflect a single representative biological replicate and the bar graphs depict the mean of replicates +/−SD (n = 3). Unless otherwise noted, significance reflects the difference between the treatment group and the media only control. *P < 0.05, *P < 0.01, ***P < 0.001.

**Figure 3:**
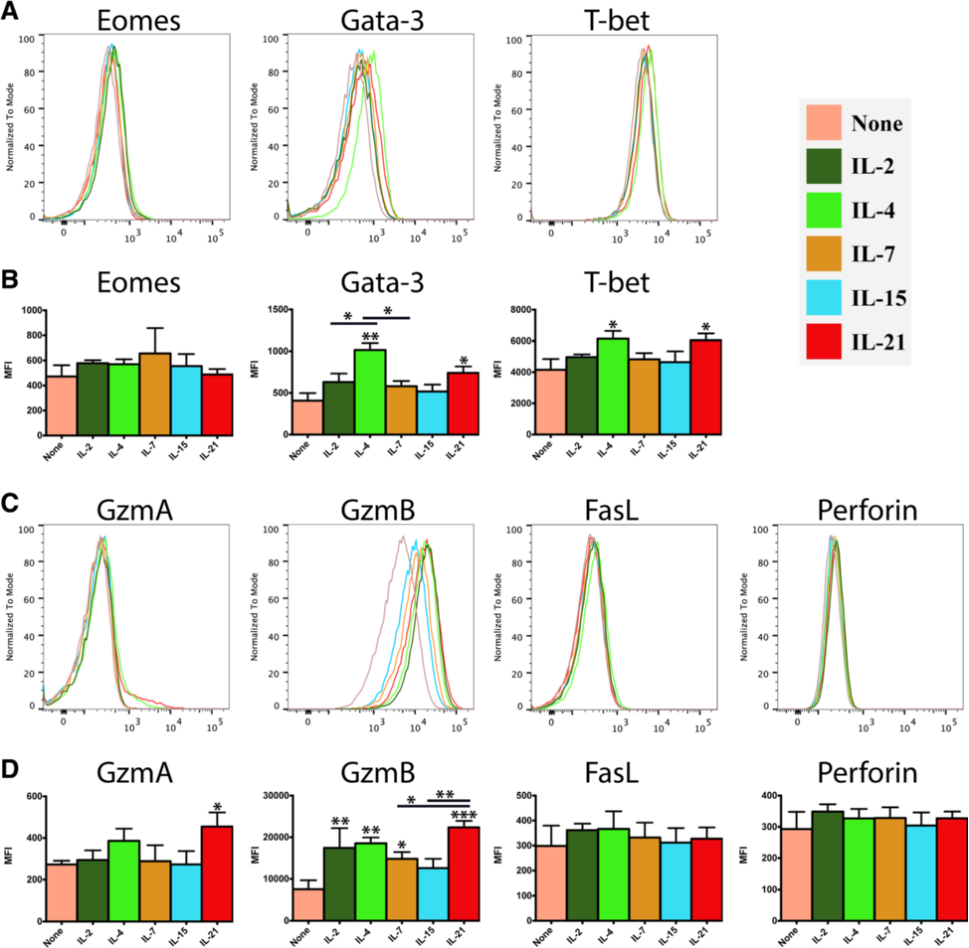
**Impact of γc cytokines on the production of transcription factors and cytotoxic effector proteins in recently-activated CD8**^**+**^**T cells.** Purified naïve OT-I CD8^+^ T cells (1x10^6^/ml) were stimulated with peptide-pulsed APCs (6x10^6^/ml). After 48 hours, OT-I T cells were harvested, re-purified and re-cultured (5x10^5^/ml) in the presence of either plain media or media supplemented with IL-2, IL-4, IL-7, IL-15 or IL-21 (100 ng/ml). After 24 hours, cells were harvested and the analyzed by intracellular cytokine staining and flow cytometry. **A**, **B)** Production of transcription factors associated with T cell fate determination. **C**, **D)** Production of effector proteins associated with cytotoxic CD8^+^ T cell function. Data are generated from 3 biological replicates, histograms reflect a single representative biological replicate and the bar graphs depict the mean +/−SD (n = 3). Unless otherwise noted, significance reflects the difference between the treatment group and the media only control. *P < 0.05, *P < 0.01, ***P < 0.001.

The ligation of co-stimulatory receptors on the surface of effector CD8^+^ T cells is known to support their survival and cytotoxic activity. Four of the most thoroughly described co-stimulatory T cell receptors are 4-1BB (CD137), GITR (CD357), ICOS (CD278) and OX40 (CD134). While several of these receptors appeared moderately up-regulated by multiple γc cytokines, IL-2 treatment induced a uniquely robust and statistically significant increase in expression of each of these receptors at the protein level (Figure 2A-B). Each γc cytokine also drove a distinct response with respect to co-repressive and suppressive T cell receptors. IL-2 treatment uniquely induced a significant increase in BTLA (CD272) and an apparent, but not statistically significant, increase in CTLA-4 (CD152), relative to the control (Figure 3C-D). Meanwhile, PD-1 (CD279) was significantly up-regulated in the IL-15 and IL-21 groups, compared to the control, while expression of TIM-3 (HAVCR2), a negative regulator of Th1/Tc1 T cells, was similar at the protein level across all treatment groups and controls.

### 2.3 IL-4 and IL-21 induce a unique increase in transcription factors and effector proteins associated with cytotoxic functions

T-bet, a transcription factor associated with T cell effector function, was elevated in response to all of the γc cytokines, although this was increase was only statistically significant for IL-4 and IL-21. GATA-3 had a similar expression profile as T-bet, although it was up-regulated at substantially higher levels by IL-4, compared to the other treatment groups. These two observations were notable because T-bet and GATA-3 are known to be essential for competing phenotypic polarizations, Tc1/Th1 and Tc2/Th2, respectively [13],[14]. Eomes, a transcription factor associated with long-lived T cell populations and regulation of central vs. effector CD8^+^ T cell differentiation also appeared elevated by several γc cytokines, but these increases did not reach statistical significance (Figure 3A-B).

The production of cytotoxic effector proteins was also differentially influenced by treatment of CD8^+^ T cells with exogenous γc cytokines (Figure 3C-D). Levels of granzyme B were significantly increased by each of the cytokines except IL-15, with the highest levels induced IL-21. Meanwhile, granzyme A production was driven primarily by IL-4 and IL-21, with only the IL-21 group reaching significance. Expression of perforin and FasL (CD178) remained relatively consistent, at the protein level, across treatment groups and controls.

### 2.4 The effect of γc cytokines on the mRNA transcriptome of recently antigen-primed CD8^+^ T cells reveals activation of distinct molecular pathways

**Figure 4:**
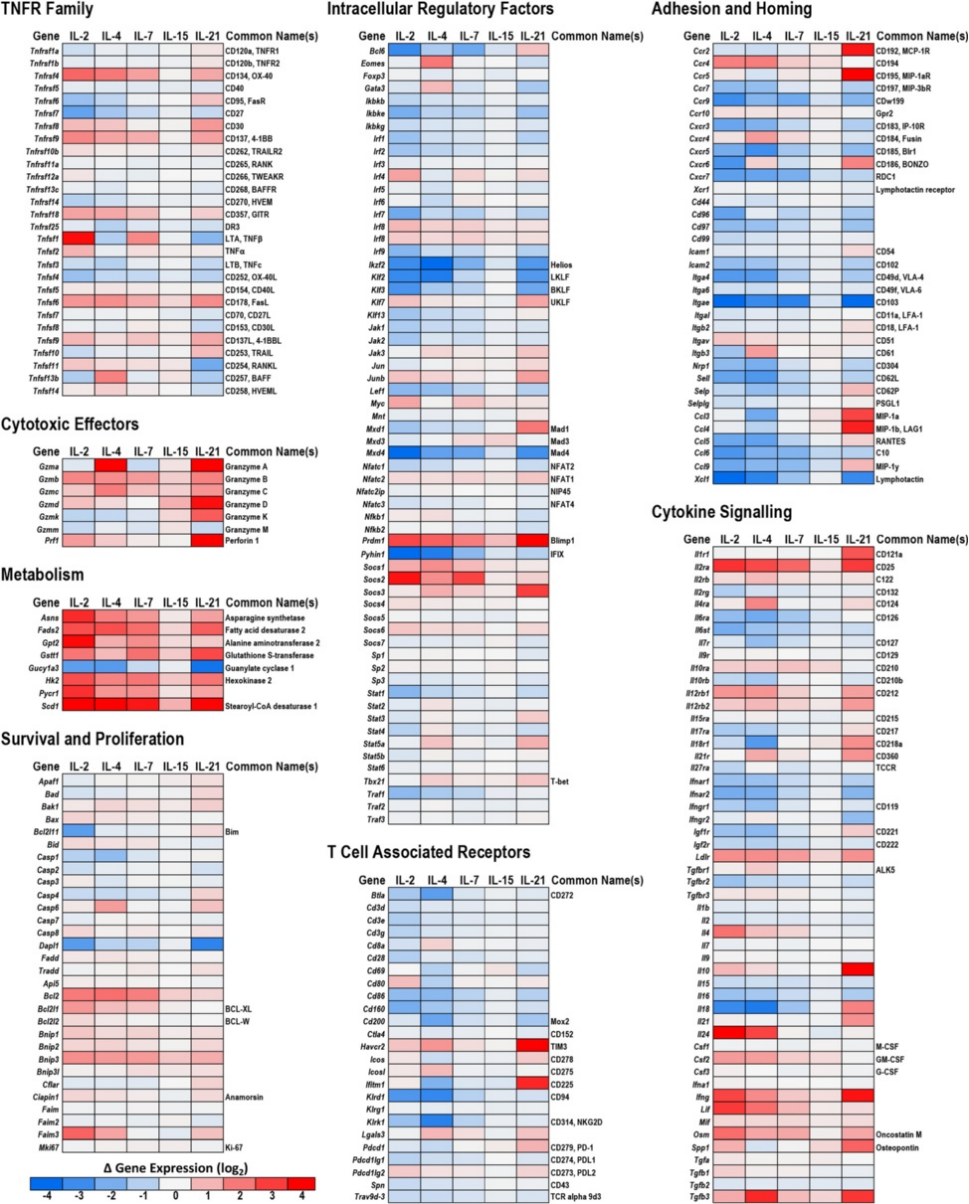
**Differential changes in the functional polarization of CD8**^**+**^**T cells induced by each γc cytokine. A-E)** Genes are grouped into functional clusters reflecting distinct biological processes associated with CD8^+^ T cells. Functional clusters each contain multiple genes, which are represented as individual dots. Up-regulation (red) and down-regulation (blue) of gene expression are represented by color, and the amplitude of the change is represented by the size of the point. Values represent the mean of three biological replicates. Lines connecting genes represent putative gene-gene interactions, as defined by the Reactome FI database. Annotation of each cluster is based on a combination of a literature meta-study and the GO annotation database. Complete gene cluster, transcriptomic data and probe information is included in Additional file 3: Table S2. **F)** 3-dimensional bubble chart illustrating the distribution of all biological replicate from each experimental condition along three functional axes: pro-survival vs pro-apoptotic, peripheral homing vs central homing, and Tc1/Th1 vs Tc2/Th2 polarization.

To better define the impact of γc cytokines on CD8^+^ T cell differentiation, and corroborate the preceding flow cytometry-based observations, the transcriptomic changes that occurred in each treatment group were profiled by mRNA/cDNA microarray. The resulting data set was filtered and sorted to highlight genes with known relevance to T cell biology and grouped into functionally and/or structurally related clusters (Figure 4, Additional file 1: Table S1A). With respect to autocrine γc signaling, the transcriptional data was consistent with observations from the cytokine bead array that showed minimal endogenous production of γc cytokines in any of the experimental groups (Additional file 2: Figure S1). In the case of IL-2, IL-4 and IL-21, exposure to those cytokines induced transcriptional up-regulation of their corresponding receptors, potentially supporting a positive feedback loop for signaling by these cytokines. However, at the protein level, evidence of positive feedback was only observed for the IL-2 receptor, CD25. Consistent with previous reports about recently-activated CD8^+^ T cells, the transcriptomic data indicated that the IL-9 receptor, CD129, was not substantially expressed in any of the conditions tested, and that the IL-15 receptor, CD215, was expressed at a significantly lower level than the other γc cytokine receptors. In this study, IL-15 did not induce dramatic changes in any of the assessed parameters, a finding which contrasts with those of several other studies investigating the impact of IL-15 on CD8^+^ T cells [15]–[19]. The low expression levels of the IL-15 receptor in this particular CD8^+^ T cell population may account for the muted effect of IL-15 treatment, relative to the other γc cytokines.

A survey of genes associated with the regulation of cell survival highlighted a clear shift towards a pro-survival phenotype in all of the γc treatment groups, although this shift was most robust in the IL-2 group. Notably, the apoptosis facilitators Bim, Caspase-2 and Dapl1 were notably down-regulated in the IL-2 treatment group, while the apoptosis inhibitors BCL2, BCL-XL, and Bnip3 were up-regulated in each treatment group, with the greatest increase observed in the IL-2 and IL-4 groups.

The transcriptional profile of T cell co-stimulatory receptors was largely consistent with the changes observed by flow cytometry, with 4-1BB, OX40, GITR all up-regulated by each γc cytokine and the most robust responses observed in the IL-2 treatment group. In contrast to the TNFR family co-stimulatory receptors, expression of the co-repressive receptors BTLA-4 and CD160 trended down in each treatment group, while expression of CTLA-4 remained stable. Expression of both PD-1 and TIM-3 were uniquely elevated in the IL-21 treatment group.

Transcriptional profiling of the granzyme family of cytotoxic effector proteins revealed distinct expression profiles in each treatment group that may reflect a divergence in the specificity and capacity of cytotoxic function. Expression of granzyme B, while highest in the IL-2 treatment group was up-regulated in response to all the γc cytokines, while granzymes A and C were elevated primarily in response to IL-4 and IL-21. Meanwhile the up-regulation of granzyme D, granzyme K and perforin was restricted to the IL-21 group. Parallel to these observations, the expression of two primary transcription factors that support T cell cytotoxic function, PRDM1 (Blimp1) and T-bet, closely mirrored changes observed in granzyme expression. Interestingly, the expression profile of PRDM1 largely matched that of granzyme B, while the profile of T-bet matched that of granzymes A and C. This correlation may be indicative of a direct connection between the expression of these particular transcription factors and cytotoxic effector genes.

The γc cytokines also induced distinct changes in the expression of genes associated with intercellular adhesion and chemotaxis. The most striking of these changes was a dramatic down-regulation in all of the γc treatment groups of the alpha-epsilon integrin (CD103), a gene associated with both epithelial localization and suppressive T cell populations. Several receptors associated with central homing, including CD62L and CCR7 were also generally down-regulated by γc treatment. With respect to peripheral homing, a striking difference was observed between the IL-21 group and the other γc cytokines, with IL-21 inducing the substantial up-regulation of CCR2 and CCR5, both of which are chemokine receptors that help lymphocytes migrate towards sites of inflammation.

A comparison of genes associated with intracellular signaling and transcriptional regulation identified several striking differences in how each treatment modulates intracellular signal transduction. The suppressor of cytokine signaling (SOCs) proteins are negative feedback regulators of JAK/STAT signaling and their transcriptional regulation can be a useful indicator of pathway activation. SOCS2 was dramatically up-regulated in response to IL-2 and IL-7, and to a lesser extent in the IL-4 group. Meanwhile, SOCS3 was substantially up-regulated in the IL-4 and IL-21 groups, with minimal changes in the others. IL-2, IL-4 and IL-7 all induced moderate up-regulation of SOCS1 and SOCS6, while little change was observed in the expression SOCS4, SOCS5 and SOCS7.

### 2.5 Visualization of transcriptomic data in functionally-related networks illustrates the distinct polarization profile induced by each γc cytokine

To contrast the impact of each γc cytokine on the polarization of distinct CD8^+^ T cell functions, we defined clusters of genes that are directly associated with, or supportive of, specific functions and phenotypes. These gene clusters were assembled by integrating data from publicly available databases with a T cell oriented literature review of genes associated with specific lymphocyte functions. The genes comprising the functional clusters (long-lived/pro-survival, short-lived/pro-apoptosis, peripheral homing, central homing, cytotoxic effectors, co-stimulatory receptors, co-repressive receptors, NK-like markers, Th1/Tc1 markers, Th2/Tc2 markers and markers of suppressive T cells) were overlaid with the transcriptomic data generated in this study (Additional file 3: Table S2). These gene clusters, their corresponding expression data and annotated gene-gene interactions were then visualized in an effort to illustrate how each γc cytokine drives the polarization of recently-activated CD8^+^ T cells in multiple functional dimensions (Figure 4A-E). Polarization along three specific axes (Tc1/Th1 vs Tc2/Th2, Long-lived vs Short-lived, Peripheral homing vs Central homing) was also visualized in 3 dimensions for each biological replicate, to illustrate the functional clustering and consistency between samples (Figure 4F).

This analysis indicates that all of the γc cytokines drive expression of pro-survival genes and suppression of pro-apoptotic genes, with IL-2 inducing the most robust response. Overall, each of the γc cytokines suppress genes associated with central homing and up-regulate those associated with peripheral chemotaxis, although peripheral homing pathways are more dramatically induced by IL-21, compared to the other cytokines. Similarly, all of the cytokines drive expression of the cytotoxic effector cluster, but IL-21 induces expression of more of these genes, and at much higher levels. The polarization between NK-like, Th1/Tc1, Th2/Tc2 and suppressive clusters offers an intriguing perspective on the functional impact of the respective γc cytokines. While the features that delineate Th1/Tc1 and Th2/Tc2 populations are generally well-defined, the NK-like T cell population remains somewhat amorphous [20]. NK-like T cells are broadly defined as cytotoxic T cells that are major histocompatibility complex-unrestricted, surface CD3^+^, express NK-cell antigens, and rearrange their T-cell receptor [21]. There are a variety of phenotypic and functional subsets within this class, but this analysis is primarily restricted to broadly conserved NK markers, such as KLRC1 (NKG2A), KLRK1 (NKG2D), and KLRD1 (CD94). IL-2, IL-7 and IL-15 up and down-regulate a mix of genes in each cluster and do not appear to have a clear polarization bias, although they each appear to down-regulate the NK-like cluster. Meanwhile, IL-4 shows a clear polarization towards the Th2/Tc2 cluster, at the expense of NK-like features. In contrast, IL-21 drives a robust up-regulation of both the NK-like and Th1/Tc1 cluster, while leaving the Th2/Tc2 cluster largely unchanged.

## 3 Discussion

Although the systemic effects of administering exogenous γc cytokines have been investigated in both i*n vitro* and *in vivo* systems, the complexity and interdependence of the γc signaling network presents a challenge to defining the *direct* effects of each γc cytokine on specific lymphocyte subpopulations. The present study addresses that challenge by characterizing the direct influence of γc cytokines on the differentiation, survival, and co-stimulatory potential of recently-activated Ag-specific CD8^+^ T cells within an experimental system designed to limit non-target cytokine signaling and cell-cell interactions. Specifically, this study interrogated how each γc cytokine altered the short-term differentiation of recently-activated CD8^+^ T cells when administered in the absence of additional stimuli and antigen-specific re-challenge. Our observations indicate that while all of the γc cytokines drive a set of shared outcomes in this population, they each induce a unique profile of phenotypic and functional polarization. Viewed in light of previous studies, the findings from this study demonstrate the importance of timing and context on the patterns of CD8^+^ T cell differentiation induced by exposure to specific γc cytokines.

Administration of γc cytokines *in vivo* has been shown to increase a variety of circulating lymphocyte populations, including CD8^+^ T cells [18],[22]–[27]. Consistent with those findings, we observed that all of the γc cytokines tested, with the exception of IL-21, supported a significant increase in both cell survival and proliferation, compared to untreated controls. Underlying these proliferative changes, we observed that expression of the anti-apoptotic factor, BCL2, mirrored the changes in proliferation at both the protein and transcriptional levels, while several markers of T cell activation and exhaustion, including Ki-67, CD69 and KLRG1 remained unchanged. The transcription of several genes required for glucose metabolism and cell growth, such as hexokinase 2 and fatty acid desaturase 2, were also markedly up-regulated in response to all of the cytokines tested. Functional grouping of transcriptomic data into pro-survival and pro-apoptotic clusters highlighted a clear shift towards a pro-survival transcriptional program, particularly in groups treated with IL-2 and IL-4, although this shift was muted in the IL-21 group. Furthermore, several receptors of pro-apoptotic signals, such as FasR and PD-1 were down-regulated by IL-2, IL-4 and IL-7, while treatment with IL-21 increased expression of these markers. In total, these findings suggest that the increase in CD8^+^ T cell populations observed following administration of γc cytokines, particularly IL-2, may reflect not just an enhancement of metabolic and proliferative cellular activity, but also a decreased sensitivity to anti-proliferative and pro-apoptotic signals.

Previous studies have indicated that treatment with exogenous IL-21 enhances tumor infiltration and the cytotoxic function of effector CD8^+^ T cells *in vivo*[28]–[31]. In this study, we observed that IL-21 uniquely increased expression of several chemokine receptors that are important for peripheral homing, notably CCR2, CCR5 and CXCR6 [32],[33]. Conversely, IL-2, IL-4 and IL-7 uniquely elevated expression of CCR4, a chemokine receptor that is also associated with peripheral homing. A similar split was observed among chemokine ligands, with IL-21 uniquely inducing expression of the MIP family ligands (CCL3, CCL4 and CCL9), which are associated with acute inflammation and recruit a range of leukocytes [34]. All of the treatment groups showed a decrease in the expression of key genes associated with central homing (CCR7, CD62L) and homing to epithelial tissues (Itgae/CD103, CCR9). The expression of genes associated with cytotoxic function, were increased by all of the γc cytokines, although the magnitude of this increase was greatest in response to IL-4 and IL-21. Taken together, these observations suggest that each of the γc cytokines support the development of cytotoxic capabilities and the migration of recently-activated T cells into the periphery, although not necessarily to epithelial tissues. However, IL-21 treated cells appear primed to follow a distinct chemokine gradient mediated by CCR2 and CCR5, one that is more likely to arise from sites of acute inflammation in the periphery [35].

Perhaps the most striking results observed in this study were the distinct differences in the polarization of CD8^+^ T cells along the traditional Tc1/Th1 and Tc2/Th2 axes [8],[10],[36],[37]. Tc1 and Tc2 are designations of CD8^+^ T cell subsets that mirror those for Th1 and Th2 CD4^+^ T cells, respectively [38]. Tc1 polarized CD8^+^ T cells are generally capable of secreting abundant IFN-γ in response to TCR activation, whereas Tc2 cells generally secrete little IFN-γ but significant amounts of IL-4, IL-5, and IL-10 in response to the same stimuli [9],[38]. Tc1 and Tc2 cells are also reported to employ distinct complements of chemokine receptors, with Tc1 cells expressing abundant CCR5, while Tc2 cells express abundant CCR4 [39]. Both subsets have been shown to have proliferative and cytolytic capacities, although these attributes tend to vary greatly between experimental models [40]–[43]. CD8^+^ T cells, at various stages of differentiation, have been polarized into Tc1 and Tc2 subsets by treatment with several different cytokine cocktails. For example, CD8^+^ T cells can be polarized into Tc1 cells by a combined treatment with IL-12, IFN-γ and an IL-4 neutralizing antibody. In contrast, these same cells can be driven to a Tc2 phenotype by treatment with IL-4 and blockade of IL-12 and IFN-γ [44]. Overall, we observed that IL-2 induced a Tc1/Th1 phenotype, IL-4 induced a Tc2/Th2 phenotype, and IL-21 induced a combined Tc1/Th1 and NK-like phenotype, while IL-7 and IL-15 did not have a preferential impact on polarization. Consistent with numerous previous reports, IL-4 treatment induced a substantial increase in GATA-3 expression, a transcription factor associated with a Tc2/Th2 phenotype [45]–[48]. The change in GATA-3 expression was accompanied by a sharp increase in transcription among genes associated with Tc2/Th2 functionality, notably CCR4 and IL-24.

Interestingly, IL-4 treatment also induced an increase in T-bet expression, which is generally associated with Tc1 polarized effector cells that produce IFN-γ following TCR stimulation [49],[50]. IFN-γ itself is also known to induce T-bet expression in lymphocytes and myeloid cells [51]. In context of the relatively promiscuous expression of T-bet across among lymphocyte subsets, the observation here that both GATA-3 and T-bet are increased in recently-activated CD8^+^ T cells may be evidence that polarization between Th1/Tc1 and Th2/Tc2 is more dependent on the balance between T-bet and GATA-3, than on the expression of the individual transcription factors. An alternative possibility is that the observation regarding T-bet expression may simply be a short-term event that does not persist through the developmental process.

**Figure 5:**
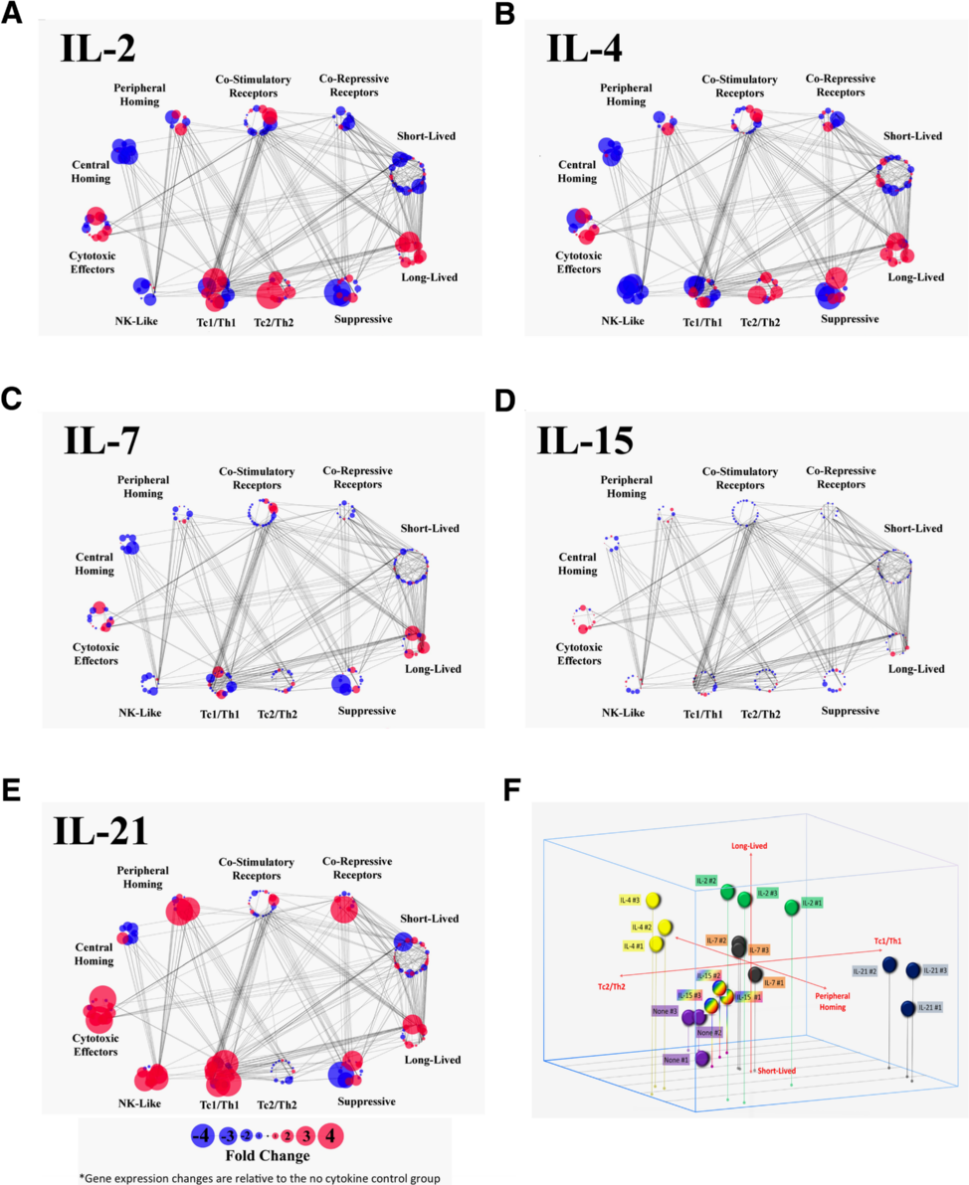
**Transcription heat maps for selected gene families associated with CD8**^**+**^**T cell differentiation and functionality.** Purified naïve OT-I CD8^+^ T cells (1x10^6^/ml) were stimulated with peptide-pulsed APCs (6x10^6^/ml). After 48 hours, OT-I T cells were harvested, re-purified and re-cultured (5x10^5^/ml) in the presence of either plain media or media supplemented with IL-2, IL-4, IL-7, IL-15 or IL-21 (100 ng/ml). After 24 hours, cells were harvested, mRNA isolated and gene expression analyzed by microarray. Data are generated from 3 biological replicates. Complete gene and probe information is included in Additional file 1: Table S1.

In contrast, IL-21 induced a dramatic shift away from a Tc2/Th2 orientation and towards a Tc1/Th1 phenotype, adopting a profile that has been described as “NK-like”. CD8^+^ T cells with an NK-like phenotype display several surface markers normally restricted to NK populations, including members of the KLRG and KLRK killer cell lectin-like receptor families, and express several distinct transcription factors [52]–[55]. NK-like CD8^+^ T cells have also been described as short-lived cells with expanded cytotoxic capabilities [56]. It is not entirely clear precisely which features define this population and what drives their formation, but it has been suggested that high levels of IFN-γ can induce this phenotype, and indeed expression of several markers of IFN-γ exposure, such as the IFITM family of interferon-inducible transmembrane proteins (IFITM1, IFITM2, and IFITM3) were substantially increased in the IL-21 treatment group [57]. However, expression of IFN-γ by the CD8^+^ T cells was broadly, and relatively evenly, up-regulated across the treatment groups, suggesting that the mechanism may involve an altered sensitivity to IFN-γ or an alternative signaling pathway, rather than a direct response to increased concentrations of IFN-γ. Alternatively, several studies have shown that CD8^+^ T cells can acquire NK-associated markers and MHC-unrestricted cytolytic capabilities via treatment with various cytokines, including IL-7, IL-12, IL-15 and IL-21[15],[55],[56],[58]. However, the inconsistent phenotypic outcomes reported by these studies, as well as the observations from this study, suggest that the capacity of specific cytokines to induce an NK-like polarization in CD8^+^ T cells may be largely defined by context and timing. While IL-4 and IL-21 drove divergent polarizations along the Tc1/Th1 vs Tc2/Th2 axis in our model, the response to IL-2, IL-4 and IL-15 was much more balanced. IL-2 has been previously reported to support both Th1 and Th2 differentiation, at the expense of Th17 differentiation [59]. However, IL-2 is also known to support the overall expansion of lymphocyte populations, irrespective of their functional orientation. In this model, we observed that IL-2 did indeed primarily induce a balanced increase in the expression of gene clusters associated with both Th1/Tc1 and Th2/Tc2 polarizations. Underlying these changes may be a positive feedback mechanism that augments the signal strength of specific γc cytokines. In this model, we observed that treatment with IL-2, IL-4 and IL-21 each significantly increased expression of their cognate receptor, which may increase the sensitivity to each respective cytokine (Figure 5, Additional file 2: Figure S1). In an *in vivo* system that contains myriad competing signals, this feedback system may be important for facilitating a robust polarization in response to small changes in cytokine concentration.

Numerous agonistic monoclonal antibodies that target TNFR family receptors are undergoing pre-clinical and clinical trials as immunotherapies for cancer [12],[60]. Several studies have reported findings that suggest administration of exogenous γc cytokines may augment the efficacy of mAb therapies that target TNFR receptors. Specifically, co-administration of IL-2 has been reported to enhance the anti-tumor immune responses of therapies targeting OX40 (CD134), GITR (CD357) and 4-1BB (CD137) [5],[6],[61],[62]. In this study, we observed that exposure to IL-2 led recently-activated CD8^+^ T cells to substantially increase their expression of 4-1BB, GITR, ICOS and OX40, at both the transcriptional and protein level. The other γc cytokines also boosted the transcriptional expression of these receptors to a lesser extent, but at the protein level these increases only reached statistical significance in one case, IL-7 induced expression of ICOS. Therefore, in this model it appears that IL-2 is far more likely to enhance the capacity of recently-activated CD8^+^ T cells to respond to co-stimulatory signals, compared with other members of the γc family.

The observations reported in this study demonstrate the importance of timing for the response of activated CD8^+^ T cells to γc cytokines. A previous study by Hinrichs, et al. reported that when exogenous IL-21 was added to the culture media during antigen priming, the acquisition of effector functions and the induction of granzyme B were inhibited, while exogenous IL-2 and IL-15 had an opposite effect, although this was not maintained following adoptive transfer [63]. However, in this study we observed that IL-21 treatment produced the most robust induction of cytolytic effector function, and several other studies have reported that exogenous IL-21 enhances cytotoxic capacity of CD8^+^ T cells *in vivo*. The key difference appears to be in both the timing and context of the stimuli. With respect to context, the addition of exogenous γc cytokine during *in vitro* antigen priming with a mixed population of leukocytes will skew the balance of γc signaling in the system, but the original complement of cytokines is still present. In contrast, this model interrogates the response of CD8^+^ T cells that are isolated prior to stimulation, creating a cytokine environment that is dominated by a single γc cytokine. Although artificial, this type of isolated stimuli may reflect some aspects of the environment encountered by the freshly-activated T cells as they migrate into the periphery, where there may be fewer sources of γc cytokine production in close proximity. Additionally, differences in the timing of the γc stimuli clearly make a critical difference. A short-term burst of IL-2 production has been shown to accompany antigen-specific priming of T cells, and unusually high levels of other γc cytokines may disrupt the initial phases of rapid differentiation that occur during early activation [64]–[66]. These observations raise the possibility that, as modulators of CD8^+^ T cell-mediated immunity, γc cytokines may be more useful when administered after immunization, rather than as a simultaneous treatment. Furthermore, the rapid and dramatic up-regulation of multiple TNFR family co-stimulatory receptors in response to IL-2 treatment suggests that this γc cytokine may be uniquely well-suited for co-administration with agonistic antibodies targeting these receptors, particularly 4-1BB, GITR, ICOS and OX40. Our findings also suggest that the cytotoxic activity of such a population may be further enhanced by IL-21 treatment, and indeed it has been reported that a combination of IL-21 with low dose IL-2 improves outcomes in a murine melanoma model, in part by enhancing the anti-tumor activity of CD8^+^ T cells [29].

## 4 Conclusions

Overall, this study suggests that among γc cytokines, IL-2 may be uniquely beneficial for therapeutic strategies that combine immunization with agonists of co-stimulatory T cell receptors. The data from this report, along with previous studies of γc cytokines and CD8^+^ T cell differentiation, indicate that the timing of IL-2 administration relative to immunization may play a critical role in defining the resulting CD8^+^ T cell response [67]. In this study, we found that administration of IL-2 significantly increased the proliferation, survival and the expression of co-stimulatory by CD8^+^ T cells when treatment followed antigen-priming by 48 hours. In line with previous reports, this study also found that IL-4 and IL-21 both induced a robust acquisition of cytotoxic functions in this population, albeit with dramatically different polarization profiles, Th2/Tc2 and NK-like, respectively [68]. Those observations support the hypothesis that IL-21 may be useful for promoting the cytotoxic function of peripheral CD8^+^ T cells, but because exogenous γc cytokine treatment may disrupt antigen-priming, only after a population of activated, tumor-reactive CD8^+^ T cells is well-established.

## 5 Methods

### 5.1 Mice

Wild-type C57BL/6 mice were purchased from Jackson Labs (Bar Harbor, ME). OT-I Thy1.1 TCR Tg mice were bred in our facility. All mice were maintained under specific pathogen-free conditions in the Providence Portland Medical Center animal facility. Experimental procedures were performed according to the National Institutes of Health Guide for the Care and Use of Laboratory Animals.

### 5.2 Cell isolation and preparation

Spleens were harvested from both wild-type C57BL/6 and OT-I Thy1.1 TCR Tg mice and processed to obtain single cell suspensions. ACK lysing buffer (Lonza, Walkersville, MD) was added for 5 min at RT to lyse red blood cells. Cells were washed and re-suspended in complete RPMI 1640 (Lonza) cell culture media supplemented with 10% FBS (Lonza), HEPES, non-essential amino acids, sodium pyruvate (all from Lonza), and pen-strep glutamine (Invitrogen). Whole splenocytes from wild-type C57BL/6 mice were used as stimulator cells and CD8^+^ T cells purified from OT-I splenocytes using the Dynal mouse CD8^+^ T cell negative isolation kit (Invitrogen) were used as antigen-specific effector cells. Purified naïve wild-type OT-I (1 × 10^6^/well) were stimulated with OVA peptide (SIINFEKL) pulsed (5 μg/ml) and irradiated (2,000 rads) syngeneic splenocytes (6 × 10^6^/well) in 24-well plates. Forty-eight hours later, activated OT-I T cells were harvested and viable cells were enriched over a Ficoll-paque gradient and washed prior to being re-seeded in media (5 × 10^5^ cells/ml) and treated with the γc cytokines. Recombinant murine IL-2, IL-4, IL-7, IL-15 and IL-21 were purchased from eBioscience (San Diego, CA). Experimental samples were treated with 100 ng/ml of their respective gamma chain cytokine and incubated at 37°C for 24 hours.

### 5.3 Cell proliferation and survival assays

The total number of viable cells post-treatment was measured by counting, using trypan blue staining to exclude dead cells. The viable proportion of CD8^+^/Thy1.1^+^ cells in each treatment group was quantified by flow cytometry, using the eFluor780 viability dye (eBioscience). The cell proliferation assay was conducted with three biological replicates.

### 5.4 Flow cytometry

The cells were pre-treated with a blocking mixture of anti-CD16/32 and a mixture of polyclonal mouse and rat IgG for 10 min at 4°C, then stained for 30 min at 4°C with the following cytokines: CD8 BV785, Viability Dye eFluor780, Thy1.1 eFluor450, Dump (CD11b, CD11c, CD19) AF70, Granzyme-A APC, Granzyme-B PE, Perforin FITC, Gata-3 eFluor710, T-bet PE-Cy7, BCL2 PE, Ki-67 FITC, Eomes eFluor710, BTLA APC, OX40 PE, 4-1BB eFluor710, GITR PE-Cy7, ICOS PerCP5.5, KLRG1 APC, TIM-3 PE, CTLA-4 FITC, PD-1 PE-Cy7, CD215 APC, CD124 PE, CD127 APC, CD25 FITC, FasL eFluor710, and CD69 PE-Cy7. All antibodies were obtained from eBioscience (San Diego, CA), BD Biosciences (San Jose, CA), BioLegend (San Diego, CA), Miltenyi Biotec (Bergisch Gladbach, Germany), or Invitrogen. For intracellular staining, cells were fixed and permeabilized with the Foxp3 Staining Buffer Set (eBioscience) according to the manufacturer's instructions. Flow cytometry data was acquired on a LSRII flow cytometer using FACSDiva software (BD Biosciences). Data was processed and visualized with FlowJo (Treestar, Ashland, Oregon). OneComp beads (eBioscience) were used to define compensation settings. Analyses were gated on viable/Thy1.1^+^/CD8^+^/Dump^−^ cells. Histograms were normalized to the mode and obtained from representative samples. Mean fluorescent intensities (MFI) was calculated using the mean MFI for each target data sets representing three biological replicates. Data sets were collated and imaged with Prism (GraphPad Software, San Diego, CA), and statistical significance was determined by one-way ANOVA.

### 5.5 RNA isolation and microarray

RNA was isolated using either the RNeasy Mini kit (Qiagen, Valencia, CA) or a combination of TRIzol reagent (Life Technologies, Grand Island, NY, Grand Island, NY) and the Direct-zol RNA miniprep kit (Zymo Research, Irvine, CA), all following manufacturer protocols. Two biological replicates for mRNA analysis were prepped using the RNeasy kit. The third replicate was prepped using the Trizol/Direct-zol approach. Prior to submission to the Oregon Health & Science University Gene Microarray Shared Resource core facility (Portland, Oregon), the purity and quantity of the RNA was assessed by NanoDrop ND-1000 (Thermo Scientific, Wilmington, DE). The quality and quantity of RNA samples was further analyzed on the Bioanalyzer platform (Agilent, Santa Clara, CA). Labeled target cDNA was prepared from total RNA samples using the Ambion MessageAmp Premier protocol (3’IVT assay). Each sample target was hybridized to a Mouse 430 2.0 GeneChip array (Affymetrix, Santa Clara, CA). Image processing and expression analysis were performed using Affymetrix GeneChip Command Console (AGCC) v. 3.1.1 and Affymetrix Expression Console v.1.1 software, respectively. Data from all biological replicates and conditions was imported into the Affymetrix Expression Console and normalized (RMA), annotated and exported to CSV files, which were then processed in Excel. Raw expression data is represented on a log_2_ scale and data from biological replicates was averaged when comparing differences between experimental conditions.

### 5.6 Gene expression data processing and analysis

A log_2_ scale was used when representing raw gene expression values, and the relative changes between experimental conditions. Averages of the biologic replicates for each condition were used when comparing changes in gene expression. The raw data for each replicate, treatment averages, comparative changes, gene information and probe ID for the genes included in Figure 5 are included in Additional file 1: Table S1A. The complete CEL files were deposited in the GEO database (accession #GSE58262). Construction of functionally related gene clusters utilized data from the GO annotation database, as well as an aggregation of markers that have been reported in the literature to be associated with specific T cell functions and phenotypes. The list of genes in each cluster, along with their expression and probe data, is available in Additional file 3: Table S2. To visualize the transcriptional changes in each functional cluster, the gene clusters were imported into Cytoscape and each cluster was represented as a circle of spots, each representing a specific gene in the cluster. Previously annotated functional interactions between genes were identified and illustrated with the Reactome FI plugin. Changes in gene expression between each experimental group and the untreated control were overlaid into the network and visualized by amplitude (spot size) and direction (color; up-regulated = red, down-regulated = blue).

### 5.7 Cytokine bead array

Supernatants from each treatment group and controls were harvested and the concentration of cytokines was measured by cytokine bead array via flow cytometry, following manufacturer protocols (eBioscience).

## Competing interests

The authors declare that they have no competing interests.

## Authors’ contributions

MM and WR conceived and designed the study and experiments. MM, MK, SL, CD, and WR performed experiments and analyzed data. MM, CD, and WR wrote and revised the manuscript. All authors read and approved the final manuscript.

## Grant support

NIH 5R00CA136678 (WLR), NIH 5T32AI078903 (MJM), and the Providence Portland Medical Foundation.

## Additional files

## Supplementary Material

Additional file 1: Table S1. Complete gene set and probe information. Click here for file

Additional file 2: Figure S1. Autocrine γc cytokine signaling by recently activated CD8 T cells treated with exogenous γc cytokines. Purified naïve OT-I CD8^+^ T cells (1x10^6^/ml) were stimulated with peptide-pulsed APCs (6x10^6^/ml). After 48 hours, OT-I T cells were harvested, re-purified and re-cultured (5x10^5^/ml) in the presence of either plain media or media supplemented with IL-2, IL-4, IL-7, IL-15 or IL-21 (100 ng/ml). After 24 hours, cells and supernatants were harvested and the analyzed. A) Secretion of cytokines into the culture supernatant. B, C) Flow cytometry analysis of the expression of specific γc receptors. Data are generated from 3 biological replicates, histograms reflect a single representative biological replicate and the bar graphs depict the mean of replicates +/−SD (n = 3). Unless otherwise noted, significance reflects the difference between the treatment group and the media only control. *P < 0.05, *P < 0.01, ***P < 0.001. Click here for file

Additional file 3: Table S2. Complete gene cluster, transcriptomic data, and probe information. Click here for file
