# Supplementary material for: Common gamma chain (γc) cytokines differentially potentiate TNFR family signaling in antigen-activated CD8+ T cells
Source: J Immunother Cancer. 2014 Sep 16;2:28. doi: 10.1186/s40425-014-0028-y (PMC4236884; doi:10.1186/s40425-014-0028-y)
Supplement: Additional file 2: Figure S1. — Autocrine γc cytokine signaling by recently activated CD8 T cells treated with exogenous γc cytokines. Purified naïve OT-I CD8+ T cells (1x10^6^/ml) were stimulated with peptide-pulsed APCs (6x10^6^/ml). After 48 hours, OT-I T cells were harvested, re-purified and re-cultured (5x10^5^/ml) in the presence of either plain media or media supplemented with IL-2, IL-4, IL-7, IL-15 or IL-21 (100 ng/ml). After 24 hours, cells and supernatants were harvested and the analyzed. A) Secretion of cytokines into the culture supernatant. B, C) Flow cytometry analysis of the expression of specific γc receptors. Data are generated from 3 biological replicates, histograms reflect a single representative biological replicate and the bar graphs depict the mean of replicates +/−SD (n = 3). Unless otherwise noted, significance reflects the difference between the treatment group and the media only control. *P < 0.05, *P < 0.01, ***P < 0.001. [file s40425-014-0028-y-S2.pdf]

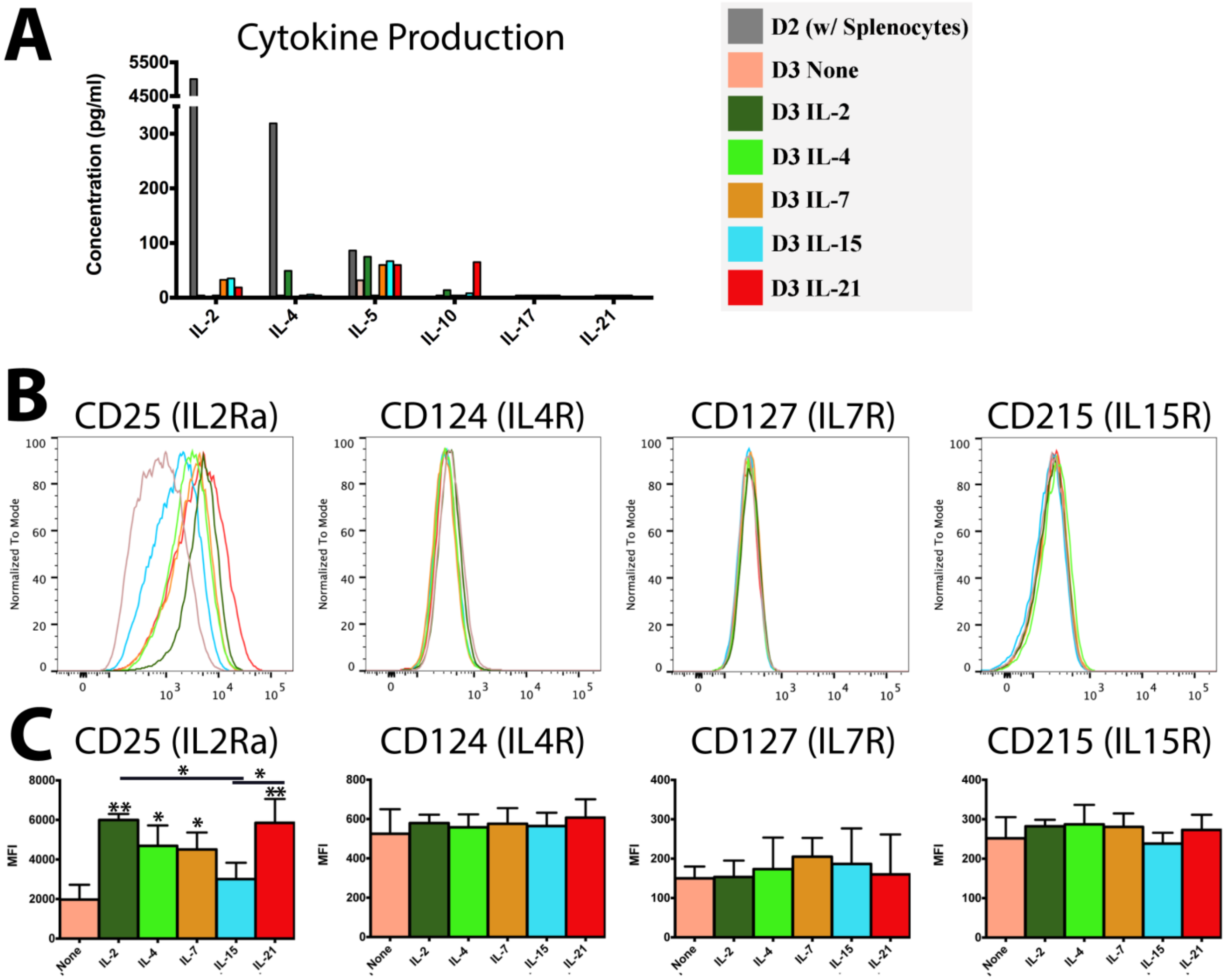

Supplemental Figure 1. Autocrine γc cytokine signaling by recently activated CD8 T cells treated with exogenous γc cytokines. Purified naïve OT-I $CD8^+$ T cells ($1x10^6$/ml) were stimulated with peptide-pulsed APCs ($6x10^6$/ml). After 48 hours, OT-I T cells were harvested, re-purified and re-cultured ($5x10^5$/ml) in the presence of either plain media or media supplemented with IL-2, IL-4, IL-7, IL-15 or IL-21 (100 ng/ml). After 24 hours, cells and supernatants were harvested and the analyzed. A) Secretion of cytokines into the culture supernatant. B, C) Flow cytometry analysis of the expression of specific γc receptors. Data are generated from 3 biological replicates, histograms reflect a single representative biological replicate and the bar graphs depict the mean of replicates +/- SD (n=3). Unless otherwise noted, significance reflects the difference between the treatment group and the media only control. *P<0.05, *P<0.01, ***P<0.001.
